# Course of mental symptoms in patients with stress-related exhaustion: does sex or age make a difference?

**DOI:** 10.1186/1471-244X-12-18

**Published:** 2012-03-12

**Authors:** Kristina Glise, Gunnar Ahlborg, Ingibjörg H Jonsdottir

**Affiliations:** 1 Institute of Stress Medicine, Carl Skottsbergsgata 22B, SE-413 19, Gothenburg, Sweden; 2 Occupational and Environmental Medicine at the Department of Public Health and Community Medicine, Sahlgrenska Academy at the University of Gothenburg, Gothenburg, Sweden

**Keywords:** Stress, Exhaustion, Burnout, Sex, Age

## Abstract

**Background:**

Long-term sick leave due to mental health problems, especially among women, is a substantial problem in many countries, and a major reason for this is thought to be psychosocial stress. The recovery period of different patient groups with stress-related mental health problems can differ considerably. We have studied the course of mental health symptoms during 18 months of multimodal treatment in relation to sex and age in a group of patients with stress-related exhaustion.

**Methods:**

The study group includes 232 patients (68% women) referred to a stress clinic and who fulfilled the criteria for Exhaustion Disorder (ED). The majority also fulfilled diagnostic criteria for depression and/or anxiety; this was similar among women and men. Symptoms were assessed at baseline, three, six, 12 and 18 months by the Shirom-Melamed Burnout Questionnaire (SMBQ) and the Hospital Anxiety and Depression scale (HAD). A total SMBQ mean score of ≥ 4 was used to indicate clinical burnout, which correlates well with the clinical diagnosis of ED.

**Results:**

There were no statistically significant differences between women and men or between young and old patients in the self-reported symptoms at baseline. The proportion that had high burnout scores decreased over time, but one-third still had symptoms of clinical burnout after 18 months. Symptoms indicating probable depression or anxiety (present in 34% and 65% of the patients at baseline, respectively) declined more rapidly, in most cases within the first three months, and were present only in one out of 10 after 18 months. The course of illness was not related to sex or age. The duration of symptoms before seeking health care, but not the level of education or co-morbid depression, was a predictor of recovery from symptoms of burnout after 18 months.

**Conclusions:**

The course of mental illness in patients seeking specialist care for stress-related exhaustion was not related to sex or age. The burden of mental symptoms is high and similar for men and women, and at the 18 month follow-up, one-third of the study group still showed symptoms of burnout. A long duration of symptoms before consultation was associated with a prolonged time of recovery, which underlines the importance of early detection of stress-related symptoms.

## Background

Long-term sick leave due to common mental health problems e.g. adjustment disorders, clinical burnout/exhaustion, anxiety and depression is a substantial problem in many countries, and a major reason for this is thought to be psychosocial stress [1,2]. These problems may be long-lasting, but this varies greatly depending on the severity and duration of the condition, characteristics of the patient and the availability of adequate treatment [3]. Patients who fulfil the criteria for adjustment disorder have been shown to have good prognosis, with the majority of patients returning to work after only three months of treatment [1]. These patients are regarded as having a less severe condition compared to those with, for example, clinical burnout and stress-related exhaustion, mainly due to higher mental burden of illness in the latter cases with co-occurrence of depression and anxiety [2,4]. The recovery period for different patient groups could thus differ considerably [2]. However, studies which examine the course of illness with focus on the mental symptoms in patients with these types of conditions are few [5,6]. It is also unclear which factors predict the course of illness. Sex may be one such factor, but the main focus in this regard has been on prevalence [4,7]. For example, burnout or exhaustion as a consequence of prolonged psychosocial stress, mainly studied in working populations, is commonly found to be higher for women compared to men [8-10]. Depression is also commonly more prevalent among women [11,12]. Another factor of possible importance for the course of illness is age; in patients with chronic musculoskeletal pain undergoing multidisciplinary rehabilitation young age was related to a favourable outcome [13]. Besides having biological implications, age is a social indicator related to work and family life. The probability of developing depression was not affected by age in a large study of depression across 29 countries [11], while another study reported higher prevalence of depression with increasing age [14]. Previous studies do not seem to assign major importance to age as a predictor of recovery in patients with depression [15] or employees on sick leave due to fatigue [16]. To the best of our knowledge, no study has explored how sex and age are related to the course of illness in patients with exhaustion due to long-term stress exposure. Clinical experience asserts that the course of illness for patients with clinical burnout/exhaustion is often long-lasting. It is thus of utmost importance to explore the course of illness in this group of patients and to identify possible factors, including sex and age, that could be of significance in explaining the long-lasting symptoms. Such knowledge is valuable for professionals such as general practitioners, occupational health practitioners, human resources professionals and social security office workers for making realistic prognoses regarding recovery and return to work, and for offering adequate treatment for a sufficient duration.

The main aim of this study was to explore the course of illness (primarily symptoms of burnout) for 18 months among patients diagnosed and treated for stress-related exhaustion. We also aimed to see whether course of illness was related to sex and age. Baseline data regarding socio-demographic factors, co-morbidity and duration of symptoms before seeking medical care, as well as use of antidepressant medication, were also evaluated as predictors of recovery.

## Methods

### Participants

The present study consists of 232 patients (158 women and 74 men), remitted from primary health care centres or occupational health service centres between 2004 and 2008 to a specialist clinic, which exclusively treats patients with stress-related mental disorders, in the region of Västra Götaland, Sweden. The patients were ambulatory at the time of the study, and none had received in-patient care due to their illness. The referral criteria were stress-related exhaustion with no apparent somatic disorder or abuse that could explain the exhaustion, and a maximum duration of ongoing sick leave of six months. Some kind of treatment had usually been initiated by the remitting physician; for example, 29% (*n *= 67) were on antidepressants (AD) at the time of referral to the specialist clinic. The physicians at the clinic initiated antidepressant treatment in additional patients; thus 70% (*n *= 162) of the patients were using AD at the six-month follow-up, and after 18 months, 57% were still on AD. At the first consultation, 62% were on full-time sick leave and 19% on part-time sick leave.

### Inclusion criteria

Only patients who fulfilled the diagnostic criteria for Exhaustion Disorder (ED, Table 1) were included in the present study. These criteria were established by the Swedish National Board of Health and Welfare in 2005 to improve diagnostics in cases of stress-related exhaustion/clinical burnout [17,18] and were assigned the code F43.8A of the International Classification of Diseases and Related Health Problems (ICD-10).

**Table 1:** Diagnostic criteria for stress-related Exhaustion disorder as proposed by the Swedish National Board of Health and Welfare 2005

Diagnostic criteria for Exhaustion Disorders	
**A**	Physical and mental symptoms of exhaustion with minimum two weeks duration. The symptoms have developed in response to one or more identifiable stressors which have been present for at least 6 months.

**B**	Markedly reduced mental energy, which is manifested by reduced initiative, lack of endurance, or increase of time needed for recovery after mental efforts.

**Diagnostic criteria for Exhaustion Disorders**	

**C**	At least four of the following symptoms have been present most of the day, nearly every day, during the same 2 week period:

	*1 Persistent complaints of impaired memory*.

	*2 Markedly reduced capacity to tolerate demands or to work under time pressure*.

	*3 Emotional instability or irritability*.

	*4 Insomnia or hypersomnia*.

	*5 Persistent complaints of physical weakness or fatigue*.

	*6 Physical symptoms such as muscular pain, chest pain, palpitations, gastrointestinal problems, vertigo or increased sensitivity to sounds*.

**D**	The symptoms cause clinically significant distress or impairment in social, occupational or other important areas of functioning.

**E**	The symptoms are not due to the direct physiological effects of a substance (e.g. a drug of abuse, a medication) or a general medical condition (e.g. hypothyroidism, diabetes, infectious disease).

**F**	The stress-related disorder does not meet the criteria for major depressive disorder, dysthymic disorder or generalized anxiety disorder.

### Exclusion criteria

Patients with somatic diseases, such as generalised pain, thyroid disease or vitamin B-12 deficiency or obesity, that could explain the exhaustion, were excluded along with patients with alcohol abuse or serious psychiatric diagnoses other than depression and anxiety. Special attention regarding diagnostic criteria was given to chronic fatigue syndrome [19] and fibromyalgia [20], which share some common symptoms with ED. Patients who fulfil the criteria for these diagnoses were referred to other clinics and thus not included in this study.

### Diagnostic procedures

Two senior physicians carried out a diagnostic procedure, obtaining an extended anamnesis and carrying out a physical examination. The diagnostic procedure for ED has been previously described in detail [21]. One important criterion requires that the physician, together with the patient, is able to identify one or more stressors that have been present for at least six months during which the symptoms developed. Another important criterion is that exhaustion is explained as extended fatigue and that the patient does not feel recovered after short-term rest, such as one or several nights of sleep. If the patient meets the criteria for major depressive disorder, dysthymic disorder or generalised anxiety disorder, these diagnoses are to be set first. Before consulting the physician, the patient completed a one-page Prime-MD symptom checklist [22]. Affirmative responses were followed-up by the physician in a structured interview form conforming to the criteria of the Diagnostic and Statistical Manual of Mental Disorders, Fourth Revision, for the diagnostic assessment of mood and anxiety disorders [23]. General anxiety, unspecific anxiety and/or panic disorders were classified as any anxiety disorder' in the study.

### Procedures of follow-up and treatment

All patients were offered multimodal treatment (MMT) with similar components but adapted to their individual needs during the whole follow-up period of 18 months. The patients were usually visiting the physician with an interval of four to six weeks. Physical activity and other lifestyle topics were repeatedly discussed at these visits, and the patient was encouraged to start physical activity as a part of the treatment. An eight-week stress reduction group programme was offered to all patients, as well as a two-hour lecture, teaching the patients the basics about stress and the consequences of chronic stress exposure. Employers, working colleagues and relatives were also offered to attend a short lecture regarding stress-related mental disorders. Cognitive behavioural group therapy for insomnia and/or a recommendation to visit a psychologist for individual psychotherapy were other treatment methods. At 18 months, 62% of the patients had received individual psychotherapy during the last 12 months. Patients were offered to participate in an aerobic exercise group and strength training at the clinic once a week for 18 weeks and 20% (*n *= 47) participated; the rest were recommended individual physical training. Antidepressant medication was offered or adjusted when needed. Finally, communication with the Social Insurance Office and the employer was facilitated, and about half of the patients participated in special meetings regarding the earliest possible return to work.

### Measurements

Shortly before the first visit at the clinic, all patients got a postal questionnaire to fill in baseline data including age, socioeconomic determinants (marital status and education), duration of symptoms, actual sick leave and use of AD. Age was dichotomised into young participants (18-39 years; *n *= 86) and old participants (40-66 years; *n *= 146). Marital status was defined as married/living together or being single. Education was defined as high or low, with at least one year college education or more defined as high education. The patients were asked to estimate how many years they had experienced having the stress-related symptoms before they sought medical help. Symptom duration was dichotomised into less than one year or one year or more. Sick leave was defined as full-time (not working), part-time (working 75%-25% of ordinary work hours) or none (working ordinary work hours), and AD medication was dichotomised into users and non-users. Treatment follow-up with questionnaires measuring symptoms of burnout, depression and anxiety was performed after three, six, 12 and 18 months. The course of illness was determined as the proportion of patients who scored above the respective cut-off for each of the three symptom scales described below.

The Shirom-Melamed Burnout Questionnaire (SMBQ) was used to measure burnout according to Melamed and co-workers [24]. The scale consists of 22 statements, and the responses are recorded on a seven-point Likert scale varying from one ('almost never') to seven ('almost always'). The total mean score can thus range from one to seven. Mean scores were calculated for the total SMBQ burnout score. In healthy workers, SMBQ has been shown to be positively associated with subjective measures of health and objective measures of physiological risk factors for cardiovascular disease [24]. It was found to correlate highly with the emotional exhaustion subscale of the Maslach Burnout Inventory and with the Pines Burnout Measure [25]. A mean score above 3.75 on SMBQ total score has been used as a cut-off to define high burnout based on quartile splits [25] and Stenlund and co-workers reported the mean score of the total scale in patients with clinical burnout to be 5.7 for women and 5.6 for men [26]. In this present study, a total mean score of ≥ 40 was used as the cut-off for burnout. This was based on clinical experience and agreed upon by experts at three national stress research clinics including our institute. Since ED and burnout are closely related conditions, we expected almost all patients to score above this cut-off during the first visit to the clinic. This makes it the primary measure of the course of illness. Cronbach's alpha for total score of SMBQ was found to be 0.90.

The widely used Hospital Anxiety and Depression scale (HAD) was chosen for assessing self-reported symptoms of depression and anxiety. It was originally developed for non-psychiatric clinics to detect states of depression and anxiety [27]. The scale consists of 14 statements concerning feelings during the past week, seven for each of the two subscales. Four response alternatives (scored 0-3) indicating degree or frequency were available for each statement. A sum score above 10 was used in this study to define symptoms of depression and anxiety, respectively [27]. HAD was found to have satisfactory factor structure and internal consistency, as well as acceptable discriminant and concurrent validity [28]. It has also been shown to be sensitive in reflecting changes over time in response to different interventions [29]. In this study, the Cronbach alpha for HAD subscale depression was 0.82 and for the subscale anxiety it was 0.81, thus indicating fairly good internal consistency.

### Baseline characteristics

The mean age for the total group was 42.9 years (SD 9.3; range 22-64), women 43.4 (SD 9.6; range 22-64), and men 41.9 (SD 8.6; range 24-60). Seventy three per cent of all participants were married, similar among women and men (Table 2). College education or more was reported by 73% of all participants compared with 32% of the general population in Sweden aged 25-64 years [30]. Forty two per cent reported symptom duration of less than one year, similar among women and men. No differences between the age groups were seen regarding level of education, self-reported duration of symptoms or use of AD. Co-morbidity of depression and/or anxiety was allowed and screened for. Nine per cent were diagnosed with ED only, while 67% had ED in combination with depression and any anxiety disorder. Thirteen per cent were diagnosed with ED in combination with depression (ICD-10 F32.0, F32.1), and the remaining 11% had ED and any anxiety disorder (ICD-10 F41.9, F41.1, F41.0). This pattern was similar for women and men, but the old patients seemed to have more co-morbid depression (18%) than the young ones (6%). The latter group, however, reported more anxiety (16% versus 8%). Fulfilling diagnostic criteria for both conditions was equally common, 68% among the young and 66% among the old patients.

**Table 2:** Baseline characteristics of patients with stress-related Exhaustion Disorder

Characteristics	N^1^	Women *n *= 158	Men *n *= 74	Total *n *= 232	*p*
		**n (%)**	**n (%)**	**n (%)**	

Age years	232				0.30

-18-39		55 (35)	31 (42)	86 (37)	

-40-66		103 (65)	43 (58)	146 (63)	

Marital status	223				0.85

-Married		111 (73)	51 (72)	162 (73)	

-Single or other		41 (27)	20 (28)	61 (27)	

Education^2^	218				0.28

-higher		112 (75)	46 (68)	158 (73)	

-lower		38 (25)	22 (32)	60 (28)	

Duration of symptoms	224				0.99

**Characteristics**	***N*^1^**	**Women *n *= 158**	**Men *n *= 74**	**Total *n *= 232**	***p***

- < 1 year		64 (42)	31 (43)	95 (42)	

- ≥ year		87 (58)	42 (58)	129 (58)	

^1^Number of patients with complete data for each characteristic

^2^Higher education is one year of college education

### Ethics

The study was approved by the regional ethical review board in Gothenburg, Sweden and conducted according to the 1964 Declaration of Helsinki and only patients who consented to the use of their clinical data for research purposes were included.

### Statistics

Patterns of change in burnout were examined by plotting the frequencies of probable cases of burnout (a total mean SMBQ score ≥ 4.0) for the total group and for each sex and age group at each follow-up. Probable cases of depression and anxiety were analysed in a similar way. Mean scores with standard deviation (SD) of SMBQ are given only to allow comparison with populations in previous studies using this measure. Pearson's Chi-Square two-tailed tests with sex and age groups respectively as independent variables were performed to analyse possible differences in the proportion of cases at each follow-up. Differences in baseline data regarding marital status, education and duration of symptoms were tested for by Pearson's Chi-Square test. The level of significance was set at *p *< 0.05. The non-parametric Cocharan's Q test was used for testing change over time in the proportion of patients who scored above the cut-off on the three symptom scales. McNemar's test was then used for group-wise comparisons between two measurement points.

The association between sex and age, respectively, as independent variables and SMBQ < 4.0 as an outcome measure at 6, 12 and 18 months follow-up was first calculated by Cox regression with constant time at risk in bivariate analyses. Results are expressed as relative risk (RR) with a 95% confidence interval. Similar bivariate analyses were performed for each of the following baseline variables: clinical depression, any anxiety disorder, number of diagnoses (1-3), marital status, symptom duration, sick leave, level of education, physical activity, and use of AD. Multivariate analysis was then performed using the Cox logistic regression model including variables that showed significant associations with the outcome in the bivariate analyses (duration of symptoms) and variables that from previous research have been identified as probable predictors (level of education and clinical depression). Unreturned questionnaires and incomplete answers were treated as missing data. Imputation was only done in complementary analyses of SMBQ (see Results). The statistical package for the social sciences, IBM SPSS Statistics 19 for Windows, was used for all statistical analyses.

## Results

### Self-reported symptoms of burnout, depression and anxiety at baseline

At baseline, 93% (*n *= 180) of the patients scored ≥ 4 on SMBQ, 34% (*n *= 77) above 10 on the HAD depression subscale, and 65% (*n *= 142) above 10 on the anxiety subscale. There were no statistically significant differences between women and men in these respects. The two different age groups also showed similar proportions regarding symptoms of burnout, depression and anxiety at baseline (data not shown).

### The clinical course from baseline to 18 months of follow-up

#### A. Symptoms of burnout

**Figure 1:**
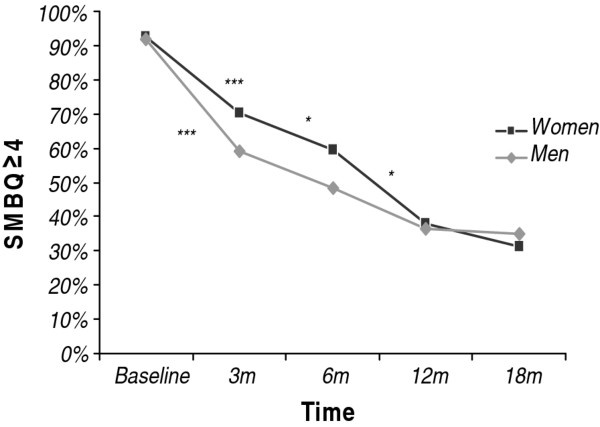
**The proportion (%) who scored ≥ 40 (mean total score) on the Shirom-Melamed Burnout Questionnaire (SMBQ) from baseline to 18 months (m) of follow-up in women (n = 128) and men (n = 66) treated for Exhaustion Disorder**. * Pairwise comparisons indicate that the difference between two adjacent time points is statistically significant (*p < 0.05 **p < 0.01 ***p < 0.001), separately for women (above line) and men (below line).

In the total group with complete SMBQ score data (*n *= 194), there was a significant decrease over time in the proportion who scored ≥ 4 between baseline and the 18-month follow-up, from 93% to 33% (*p *< 0.001). Mean SMBQ scores were 5.3 (SD 0.8), 4.4 (SD 1.2), 4.0 (SD 1.2), 3.5 (SD 1.2) and 3.2 (SD 1.3) respectively for the time points baseline, three, six, 12 and 18 months follow-up. Pairwise comparisons using the five different time points showed a significant decrease between baseline and three months (93% to 66%; *p *< 0.001), three and six months (66% to 52%; *p *< 0.001), six and 12 months (52% to 38%; *p *< 0.001), but not between 12 and 18 months (38% to 33%; *p *= 0.16). Thus, for the total group there are still 33% of patients who scored above the cut-off for clinical burnout at the 18 months follow-up. When the group was divided by sex, the same pattern was found for the women. Among men, a statistically significant decrease was seen between baseline and three months (92% to 59%; *p *< 0.001), but the differences between each three month interval thereafter did not reach statistical significance. However, the change in symptoms between three and 18 months was significant for both men and women (59% to 35%; p < 0.001 for men and 70% to 31%; p < 0.001 for women, Figure 1). There was no significant sex difference in the proportion that scored 40 on the SMBQ at any time point, including baseline.

The pattern of improvement was similar in young patients compared to old patients, and there was no significant difference between the age groups at any time point, including baseline. Both age groups showed a clear decrease between baseline and three months (92% to 64% among young and 93% to 67% among old; *p *< 0.001). Between three and six months the decrease was less prominent but still significant, young 64% to 53%; *p *= 0.05 and old 67% to 52%; *p *= 0.002.

Since there was more missing answers in SMBQ compared to the other scales used, complementary analyses were performed. Imputation of means of given answers for missing in each subscale (only one missing allowed) increased the sample size to 228 and the proportion still scoring burnout at 18 months rose to 36% compared to 33% in the originally analysed group. These analyses gave similar results as reported above and no differences were seen between men and women or different age groups (data not shown)

#### B. Symptoms of depression

**Figure 2:**
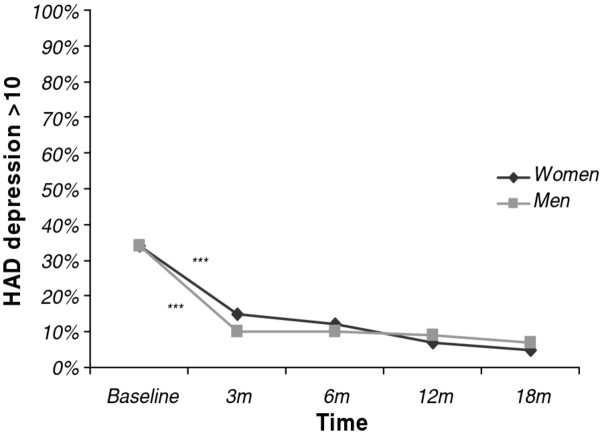
**The proportion (%) who scored > 10 on the depression subscale of the Hospital Depression and Anxiety (HAD) scale from baseline to 18 months (m) of follow-up in women (n = 154) and men (n = 71) treated for Exhaustion Disorder**. * Pairwise comparisons indicate that the difference between two adjacent time points is statistically significant (*p < 0.05 **p < 0.01 ***p < 0.001), separately for women (above line) and men (below line).

In the total group with a complete score on the HAD depression subscale (*n *= 225), there was a significant decrease over time in the proportion of patients who scored above 10 between baseline and the 18-month follow-up, (from 34% to 6%; *p *< 0.001). Pairwise comparisons using the five different time points showed a significant decrease only between baseline and three months (34% to 13%; *p *< 0.001). This applies to both women and men, when analysed separately (34% to 15% and 34% to 10% respectively; *p *< 0.001), but among women, there was also a similar trend between six and 12 months (12% to 7%: *p *= 0.08). There was no significant sex difference in the proportion that scored above the cut-off at any time point, including baseline (Figure 2). The proportion of young and old patients who scored above the threshold for depressive symptoms did not differ at any time point, including the 18 month follow-up (data not shown).

#### C. Symptoms of anxiety

**Figure 3:**
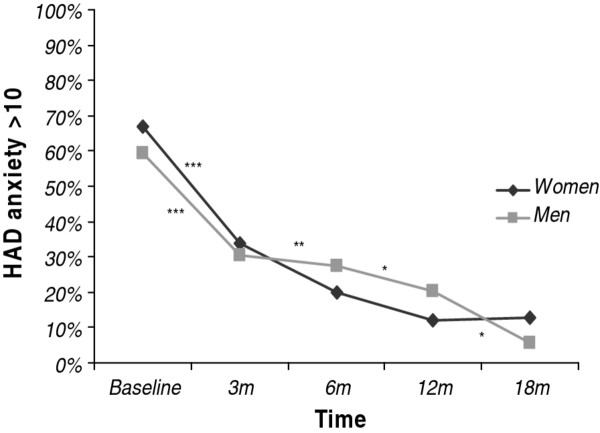
**The proportion (%) who scored > 10 on the anxiety subscale of the Hospital Depression and Anxiety (HAD) scale from baseline to 18 months (m) of follow-up in women (n = 154) and men (n = 71) treated for Exhaustion Disorder**. * Pairwise comparisons indicate that the difference between two adjacent time points is statistically significant (*p < 0.05 **p < 0.01 ***p < 0.001), separately for women (above line) and men (below line).

In the total group with complete HAD anxiety data (*n *= 220), there was a significant decrease over time in the proportion of patients who scored above 10 between baseline and the 18 month follow-up, (from 65% to 11%; *p *< 0.001). Pairwise comparisons using the five different time points showed a significant decrease between baseline and three months (65% to 33%; *p *< 0.001), three and six months (33% to 22%; *p *= 0.003), and six and 12 months (22% to 15%, *p *= 0.02) but not between 12 and 18 months (15% to 11%; *p *= 0.18). When analysed separately, both women and men showed a significant decrease between baseline and three months (67% to 34% and 60% to 30% respectively; *p *< 0.001), but the changes between three and six months (34% to 20%; *p *= 0.001) and six and 12 months (20% to 12%: *p *= 0.04) were significant only for the women and between 12 and 18 months only for the men (20% to 6%; *p *= 0.006). There was no significant sex difference in the proportion that scored above 10 on anxiety at any time point, including baseline (Figure 3). The proportion of young and old patients who scored above the threshold for symptoms of anxiety did not differ at any time point, including the 18 month follow-up (data not shown).

### Predictors for recovery of symptoms of burnout

Probable predictors for recovery of burnout (sex, age, education, symptom duration, co-morbid depression, use of AD and sick leave) were originally tested by only including the patients who scored ≥ 4 at baseline with complete data (*n *= 180). Among these, only symptom duration at baseline significantly predicted recovery. The variables included in the final model are presented in Table 3. Two complementary analyses regarding age were performed, one which divided the patients into three age groups (18-38, 39-47 and 48-64) and another which treated age as a continuous variable. None of these analyses showed any significant predictive effects of age on symptoms of burnout.

**Table 3:** Multivariate analysis^1 ^of possible predictors of recovery from symptoms of burnout during follow-up

Predictors		6 months		12 months		18 months	
	*n*^2^	RR	95% CI	RR	95% CI	RR	95% CI
Age							
-young (18-39)	67	1		1		1	
-old (40-66)	103	1.00	0.63-1.57	1.14	0.76-1.71	1.02	0.69-1.49
Sex							
-men	57	1		1		1	
-women	113	0.93	0.58-1.49	1.03	0.68-1.56	1.11	0.74-1.67
Education^3^							
-higher	121	1		1		1	
-lower	49	1.37	0.86-2.20	1.27	0.84-1.92	0.99	0.65-1.50
Symptom duration							
- < 1 year	69	1		1		1	
- > 1 year	101	0.63	0.40-0.99	0.62	0.42-0.92	0.81	0.55-1.18
Clinical depression							
-No	33	1		1		1	
-Yes	137	0.63	0.38-1.04	0.77	0.49-1.21	0.84	0.54-1.32

^1 ^Cox multivariate regression analysis of possible predictors for scoring < 4.0 (mean total score) on the Shirom-Melamed Burnout Questionnaire at six, 12 and 18 months of follow-up of patients treated for stress-related Exhaustion Disorder and who scored above that cut-off at baseline. All the variables in the table were included in the model

^2 ^Number of complete answers

^3 ^Higher education is one year of college education or more

## Discussion

Our main results show that the course of illness, measured as symptoms of burnout, depression and anxiety in patients treated with MMT for stress-related exhaustion, was not related to sex or age. The burden of mental symptoms in this group of patients is high and is similar for female and male patients. The proportion of patients who had a high score for burnout decreased gradually over time, and the most pronounced decrease is seen at the first follow-up after three months of treatment. However, as many as one-third of the patients still reported high symptom burden after 18 months, indicating a need for further treatment. At the same time of follow-up, only one out of 10 scored above the cut-off for probable depression or anxiety, indicating a rather good effect of the treatment on the co-morbid conditions. Thus, the results indicate that this patient category, characterised by a high burden of mental and psychosomatic symptoms, often needs a long time to recover from the symptoms of burnout. This is the case even when intensive, individualised MMT at a specialised clinic is offered up to 18 months. Interestingly, this seems to apply to both sexes as well as young and old patients.

The group of patients included in this study reported burnout with extensive exhaustion and co-morbid depression and anxiety. This might explain the difference in the course of illness compared to what has been seen in previous studies, for example, in patients with adjustment disorder, which is considered to represent patients with minor mental health problems, usually without other complicating co-morbid psychiatric conditions [1]. Thus, patients who seek care for stress-related mental health problems can differ considerably in respect of recovery and time needed for rehabilitation and this could partly be explained by the severity of mental health symptoms. In a four-year follow-up, a comparison between three groups from a working population showed that the group with combined burnout/fatigue problems had the most unfavourable course in terms of persistence of symptoms and absenteeism compared to individuals who reported only burnout or fatigue. The pure burnout group had the fastest rate of recovery [31]. The patient group in our study seems to be more comparable to this combined burnout/fatigue group. Thus, consideration regarding the clinical characteristics of the patient group is important when estimating plausible time for recovery.

A substantial proportion of the patients still have a high score for burnout after 12 months, and the majority of these patients are still above the selected cut-off at 18 months. More studies are needed to explore this group and the plausible reason for this long-term course of illness and lack of further improvement, at least within the time frame of this study. One plausible explanation could be ongoing stress exposure or new stressors during the course of illness and rehabilitation. Furthermore, fifteen persons out of 121 who scored below the cut-off at 12 months scored above cut-off at 18 months indicating a fluctuating course, at least in some cases.

### Men and women show a similar course of symptoms

Commonly, the prevalence of burnout and other stress-related complaints are higher among women than men. This has been shown in population studies in Sweden and other countries, using either the same burnout measure as in this present study or, for example, the Maslach Burnout Inventory [8-10]. This is also confirmed in our clinical work as about two-thirds of the patients referred to the clinic are women. However, we did not find any statistically significant difference between the sexes at any time point regarding the level of burnout or symptoms of depression and anxiety. Hence, in contrast with the large sex difference seen for the prevalence rate, women and men who seek medical care for stress-related exhaustion do not seem to differ substantially regarding the burden of symptoms. A slight sex difference in the decrease of symptoms during the 18 months follow-up was seen. Among women, there was a continuous and statistically significant decrease in symptoms of burnout from baseline to 12 months. There was a similar trend among men, but the decrease was only significant between baseline and follow-up after three months. This could be due to the small sample size for men. Recently a longitudinal study from Canada showed a disadvantage for women in remission of mental disorders [32]. Similarly, in a prospective observational cohort study of patients with fatigue in primary care, male sex was positively associated with a faster recovery [33], a finding that is not supported by our results. One possible explanation could be a difference between the patient groups included in these studies. The present study represents the most severe cases remitted from primary care with generalised symptoms of exhaustion, cognitive weariness and extensive co-morbidity. One explanation for the lack of sex difference could thus be due to the severity of symptoms, as this is similar for both male and female patients.

### Age does not predict recovery

There were no differences in symptoms of burnout, depression or anxiety at any time point measured between young and old patients in this present study, and age did not predict the recovery of burnout symptoms. It is unclear from previous literature, how and whether age predicts recovery of mental health symptoms. Similar to our data, Huibers and co-workers showed that age does not predict recovery in fatigued employees on sick leave [16]. However, other studies have shown that higher age predicts longer recovery time until return to work due to mental disorders [34]. Also, a better outcome with regard to remission was shown for young persons in a primary care study of patients treated for depression [35]. There are indications that age is related to biological changes in the response to chronic stress [36,37]. One plausible explanation for the fact that age does not matter in this study could be that the majority of the patients is between 30 and 50, and thus even if the group is divided into young and old, conceivable differences that might be present are not detected. However, we also performed additional analyses both treating age as a continuous and as a trichotomized variable, none of which showed a statistically significant association with symptom recovery regarding burnout.

### Symptom duration: A significant predictor for course of illness

While recovery of mental symptoms was not associated with sex, age, education or co-morbid depression, the patients who reported symptom duration longer than one year before seeking specialist care were less likely to score below the cut-off for burnout at six and 12 months.

Remission rates were investigated in a study of depressed outpatients commencing antidepressant therapy. Higher remission rates were correlated with a lower number of previous episodes and shorter current episode duration [35]. Our finding that the only significant predictor of course of illness in this patient group is symptom duration prior to their seeking medical care underlines the importance of early detection of patients at risk of developing stress-related exhaustion. It is quite common, in our experience, that patients with this type of health problem report that they have been experiencing symptoms for several years prior to specialist consultation. Women could be expected to seek help earlier when experiencing symptoms [36,37]. The patients often report that they have sought medical care for somatic symptoms such as chest pain, headache or gastrointestinal problems and that they received treatment for the single symptom only. Thus, screening for stress exposure in patients seeking medical help for one or several symptoms commonly related to stress should be considered as a preventive tool for avoiding long-term sick leave.

### Antidepressants

There was no difference between women and men or young and old patients in antidepressant (AD) use, and such medication did not predict the course of burnout symptoms. Clinical observation however, confirms that the patients on AD often report subjective improvement as less irritability and fewer symptoms of anxiety and depression, which could facilitate engagement and participation in other treatments included in the MMT module, and diminish family conflicts. We can, however, not conclude from this study whether AD is beneficial for patients with ED, and randomised controlled studies are needed to explore this issue.

### Methodological consideration regarding self-rating symptoms of depression and anxiety

Only 40% of the participants diagnosed with clinical depression scored above 10 on the HAD at baseline. This is in line with the previous finding from Demyttenaere and co-workers [38], showing that 66% of clinically depressed patients scored 'probable depressive disorder' on HAD. The large divergence between self-report and clinical diagnosis needs to be further studied, and it is clear that caution has to be exercised when using self-ratings of symptoms as an indicator of depression. A previous review on the validity of the HAD found the scale to perform well in assessing the symptom severity and caseness of anxiety disorders and depression in somatic, psychiatric and primary care patients and in the general population [28], but this does not seem to be the case for this patient group. If we used the cut-off of seven instead in this study, which was earlier recommended for probable depression or anxiety disorders [27], it seems to capture the clinical diagnosis in this patient group better as 81% of the patients with clinical depression scored above this lower cut-off.

### Clinical perspective

Clinicians in general practice and occupational health services who meet patients with mental health problems due to prolonged stress-exposure need to be aware that patients with symptoms of extensive exhaustion, co-morbid depression and anxiety constitute an important subgroup in need of special attention. Pronounced exhaustion together with cognitive weariness is nevertheless the core and longest-lasting symptom. Thus, treating the depression or anxiety solely is not sufficient, and the main focus should be on symptoms of burnout as these are still present in one-third of the patients after 18 months of follow-up according to this study. Mental health problems, including burnout and exhaustion, are more frequent among women, and more women are seeking help for stress-related mental health problems. However, the sex aspect of prevalence of stress-related mental health problems does not seem to apply when considering recovery of symptoms in clinically ill patients. Clinicians should therefore expect the duration of illness in this patient group to be similar for both sexes, and similar attention should be given to patients suffering from stress-related exhaustion, irrespective of age. Early detection of possible cases of stress-related exhaustion is recommended. We have recently published a study presenting a self-rating instrument for ED [38]. Individuals reporting ED who were still working were more likely to report a period of sick leave at the follow-up conducted two years later. We suggest that this instrument might be useful for both primary care and occupational health services for identifying a possible risk of future severe mental health problems.

### Limitations and strengths

Several limitations and strengths of this study need to be discussed. One major issue which needs to be highlighted is that the present study is not a randomised treatment study. We are following patients consecutively during treatment at the clinic regarding symptoms of mental health complaints. The patients are treated according to the same model but with somewhat different components, as the MMT is individually based. The decline of symptoms over time could thus to some extent depend on different treatments within the MMT model. Different groups (such as women and men) studied could have received different treatment. We did some additional analyses to see whether there were any apparent differences between women and men or young and old patients regarding which treatments were offered. No differences were seen when comparing available information in the medical records, and we have no reason to believe that the groups differ considerably regarding participation in different treatment alternatives. One major difference could be that some, but not all, patients were taking AD, but medication did not predict recovery.

There seems to be referral bias in that persons with higher education are more inclined to ask for more specialised care when consulting their general practitioner. Thus, the patients included were mostly well educated and differed in this aspect from the general population, so inference to other groups has to be done with caution. The strength of our data set is the relatively large number of patients treated at one clinic and seen by only two senior physicians. The course of mental symptoms during such a long-term follow-up as 18 months has, to our knowledge, not previously been studied in patients treated for stress-related exhaustion.

Unfortunately the largest loss of data due to missing values on single items was on SMBQ, leading to the exclusion of thirty-eight persons from those analyses. Most commonly the item "I feel no energy for going to work in the morning" was missing. This is expected since many patients were on sick leave when filling in the questionnaire. Also, severely ill patients are more likely to give incomplete answers due to their difficulties to concentrate. The total mean SMBQ score at baseline in the missing group was somewhat higher (5.6) compared to the analysed group (5.3). This could suggest that the proportion of patients not recovered from symptoms of burnout at 18 months may be even higher than our analysis show. The complementary analyses with imputation for missing values gave support to this assumption.

## Conclusions

The course of mental illness, measured as symptoms of burnout, depression and anxiety in patients seeking specialist care for stress-related exhaustion, was not related to sex or age. The burden of mental symptoms is high and similar for female and male patients, and at the 18 month follow-up, one-third of the study group still show symptoms of burnout despite extensive MMT. Long duration of symptoms before being referred to specialist treatment was associated with prolonged time of recovery, which underlines the importance of early detection of stress-related symptoms.

## Competing interests

The authors declare that they have no competing interests.

## Authors' contributions

KG has made substantial contributions to the data collection, concept, design and data analysis, and for writing the manuscript. She has also clinically examined and treated at least half of the patient group for 18 months. GA has made substantial contributions to the concept, design and data analysis, and revised the manuscript critically. IJ has made substantial contributions to the concept, design and data analysis, and revised the manuscript critically. All authors read and approved the final manuscript.

## Pre-publication history

The pre-publication history for this paper can be accessed here:

http://www.biomedcentral.com/1471-244X/12/18/prepub
